# *miR-449a* Repression Leads to Enhanced NOTCH Signaling in *TMPRSS2:ERG* Fusion Positive Prostate Cancer Cells

**DOI:** 10.3390/cancers13050964

**Published:** 2021-02-25

**Authors:** Simone Bauer, Leonie Ratz, Doreen Heckmann-Nötzel, Adam Kaczorowski, Markus Hohenfellner, Glen Kristiansen, Stefan Duensing, Peter Altevogt, Sabine M. Klauck, Holger Sültmann

**Affiliations:** 1 Division of Cancer Genome Research, German Cancer Research Center (DKFZ), German Cancer Consortium (DKTK), and National Center for Tumor Diseases (NCT), 69120 Heidelberg, Germany; simone.bauer@dkfz.de (S.B.); d.heckmann@dkfz-Heidelberg.de (D.H.-N.); s.klauck@dkfz-heidelberg.de (S.M.K.); 2 Medical Faculty, Heidelberg University, 69120 Heidelberg, Germany; 3 Department of Obstetrics and Gynecology, University Hospital of Cologne, 50937 Cologne, Germany; leonie.ratz@uk-koeln.de; 4 Computer Assisted Medical Interventions, German Cancer Research Center (DKFZ), 69120 Heidelberg, Germany; 5 Molecular Urooncology, Department of Urology, University Hospital Heidelberg, 69120 Heidelberg, Germany; adam.kaczorowski@med.uni-heidelberg.de (A.K.); stefan.duensing@med.uni-heidelberg.de (S.D.); 6 Department of Urology, University Hospital Heidelberg and National Center for Tumor Diseases (NCT), 69120 Heidelberg, Germany; markus.hohenfellner@med.uni-heidelberg.de; 7 Center for Integrated Oncology, Institute of Pathology, University of Bonn, 53127 Bonn, Germany; glen.kristiansen@ukbonn.de; 8 Skin Cancer Unit, German Cancer Research Center (DKFZ), 69120 Heidelberg, Germany; p.altevogt@dkfz-heidelberg.de; 9 Department of Dermatology, Venereology and Allergology, University Medical Center Mannheim, Heidelberg University, 68167 Mannheim, Germany

**Keywords:** prostate cancer, *TMRPSS2: ERG* gene fusion, *miR-449a*, NOTCH signaling

## Abstract

**Simple Summary:**

The reason for the frequent presence of the *TMPRSS2:ERG* (T2E) gene fusion in prostate cancer (PCa) is not fully understood. We were interested in investigating epigenomic alterations associated with the T2E gene fusion in PCa cells. Making use of publicly available genome-wide data and in vitro analyses using an LNCaP cell line model with inducible T2E expression, we uncovered a molecular network driving NOTCH signaling in T2E-positive PCa. We show that ERG directly activates *NOTCH1* and *HES1* expression by interacting with their promoter regions. Furthermore, NOTCH is activated by downregulation of *miR-449a* in *ERG* overexpressing cells. Experimental NOTCH pathway inhibition as well as *HES1* knockdown reduced oncogenic capacities in vitro, suggesting that the NOTCH pathway triggers oncogenic processes in *TMPRSS2:ERG-*positive PCa. HES1 and ERG expression are correlated in tissue samples from PCa patients. Our data suggest a novel epigenomic network driving NOTCH signaling in T2E+ PCa cells.

**Abstract:**

About 50% of prostate cancer (PCa) tumors are *TMPRSS2:ERG* (T2E) fusion-positive (T2E+), but the role of T2E in PCa progression is not fully understood. We were interested in investigating epigenomic alterations associated with T2E+ PCa. Using different sequencing cohorts, we found several transcripts of the *miR-449* cluster to be repressed in T2E+ PCa. This repression correlated strongly with enhanced expression of NOTCH and several of its target genes in TCGA and ICGC PCa RNA-seq data. We corroborated these findings using a cellular model with inducible T2E expression. Overexpression of *miR-449a* in vitro led to silencing of genes associated with NOTCH signaling (*NOTCH1*, *HES1*) and *HDAC1*. Interestingly, *HDAC1* overexpression led to the repression of *HES6*, a negative regulator of the transcription factor *HES1*, the primary effector of NOTCH signaling, and promoted cell proliferation by repressing the cell cycle inhibitor p21. Inhibition of NOTCH as well as knockdown of *HES1* reduced the oncogenic properties of PCa cell lines. Using tissue microarray analysis encompassing 533 human PCa cores, ERG-positive areas exhibited significantly increased *HES1* expression. Taken together, our data suggest that an epigenomic regulatory network enhances NOTCH signaling and thereby contributes to the oncogenic properties of T2E+ PCa.

## 1. Introduction

Prostate cancer (PCa) is the second most frequent cancer in men worldwide and accounts for approximately 4% of all cancer related deaths [1]. The gene fusion (T2E) between the gene coding for the transmembrane protease serine 2 (*TMPRSS2*) and the ETS-related gene (*ERG*) occurs in 40–50% of PCa [2] and is the most prevalent molecular alteration among all human solid tumors. In the most frequently observed rearrangement variant T2E III the fourth exon of *ERG* is fused to the 5′UTR of *TMPRSS2*, resulting in an N-truncated version of *ERG* expressed via the AR-regulated *TMPRSS2* promoter [3]. A complex interactive network of ERG, androgen receptor (AR) and different epigenetic modifiers promotes PCa progression [4,5]. However, despite its high frequency, most studies suggest that the T2E gene fusion in PCa has no prognostic relevance [6]. ERG is a master transcription factor, altering the gene expression profile of tumors harboring the gene fusion, interacting with and deregulating epigenetic modifiers, which leads to distinct DNA methylation and H3K27ac profiles in T2E positive tumors (T2E+) [2,4,7,8,9]. ERG stimulates vascularization and angiogenesis in human endothelial cells by activating NOTCH signaling or regulating super-enhancers [10,11,12]. NOTCH signaling is a highly conserved pathway promoting embryonic axis formation, epidermal differentiation, cardiac development, angiogenesis, neurogenesis, and tissue homeostasis [13,14,15,16,17,18]. There are controversies about NOTCH function in different tumor entities, suggesting that it plays a highly context-specific role [19]. In both, fusion negative and positive PCa, NOTCH can foster therapy resistance to the AR inhibitor enzalutamide or to docetaxel, and NOTCH suppression enhances the efficacy of androgen deprivation therapy [20,21]. In PCa, ERG was also reported to induce NOTCH signaling by activating histone marks in the promoter regions of NOTCH genes [9].

Given the various effects of ERG on the epigenome of tumor cells, we were interested in examining the interplay of miRNAs and mRNAs upon *ERG* overexpression in PCa. To this end, we explored The Cancer Genome Atlas prostate adenocarcinoma (TCGA-PRAD) RNA-seq data [2] to identify pathways upregulated in T2E+ PCa. Previously, we studied miRNA expression profiles [22] to obtain significantly repressed miRNAs in fusion-positive PCa. Here, we performed target gene analysis of the repressed miRNAs, followed by pathway analysis in T2E+ PCa, to identify pathways activated in PCa tissues. We verified our hypothesis of miRNA mediated signaling pathway deregulation using various PCa cellular models and tissue microarrays.

## 2. Materials and Methods

### 2.1. Sequencing Cohorts

TCGA-PRAD RNA-seq, DNA methylation (HumanMethylation450 BeadChip, Illumina, San Diego, CA, USA) and miRNA-seq data were taken from https://tcga.xenahubs.net (accessed on 30 January 2017). *ERG* expression levels from TCGA-PRAD RNA-seq data [2] were used to determine the T2E fusion status (Figure S1). GSEA (version 4.0.3, www.gsea-msigdb.org (accessed on 22 August 2019)) was performed using the T2E-separated TCGA-PRAD data on hallmark gene sets (H). For expression analysis of *miR-449a,* NAs were assumed as 0. The International Cancer Genome Consortium (ICGC-EOPC) RNA-seq data (accession number EGAS00001002923, www.ebi.ac.uk (accessed on 21 September 2020)) were taken from [23].

### 2.2. Cell Culture Methods

VCaP, PC-3, DU-145 and LNCaP cells were purchased from American Type Culture Collection (ATCC, Manassas, VA, USA), cultured and maintained according to the manufacturer’s instructions. Establishment of the LNCaP T2E #126 cells (LNCaP ev, LNCaP T2E III and LNCaP T2E VI) was described previously [24].

### 2.3. SiRNA-Mediated Gene Knockdown, miRNA Mimic Transfection and miRNA Inhibition

SiRNA mediated knockdown was performed as described previously [24]. Briefly, siRNA was transfected according to the manufacturer’s instruction (siHES1_5, siHDAC1_4, siADAM10_10, siADAM17_1) (Qiagen, Germantown, MD, USA) to a final concentration of 20 nM. The AllStars siRNA (Qiagen) was transfected as a negative control. MiRNA mimic *449a* (Dharmacon, Cambridge, UK) was transfected to a final concentration of 50 nM, with *C. elegans* miRNA cel-miR-67 (Dharmacon) as negative control (NTC). MiRIDIAN Hairpin Inhibitor (Dharmacon) and the corresponding control, were transfected to a final concentration of 50 nM using RNAiMax. Cells were harvested 72 h post transfection. In LNCaP T2E cells, doxycycline treatment for induction of T2E expression was performed for 48 h, before miRNA mimic transfection and repeated doxycycline treatment were performed for additional 72 h.

### 2.4. Pharmacological Inhibitors

Pharmacological inhibitors MK-0752 (Biomol, Germany, Hamburg), Batimastat (Biomol), 4-(dimethylamino)-N-[6-(hydroxyamino)-6-oxohexyl]-benzamide (DHOB) (Santa Cruz, Dallas, TX, USA), and tranylcypromine (TCP) (BIOTREND Chemikalien, Köln, Germany) were dissolved in DMSO. DMSO treatment only served as a negative control. Treatment was performed for 72 h.

Cell lysis and Western blots were performed in biological replicates as described previously [24]. The chemiluminescent signal was detected using the ChemiDoc XRS+ system (Bio-Rad, Munich, Germany). All antibodies used in this work are specified in Table S1.

### 2.5. Cloning Strategy and Gene Overexpression In Vitro

Cloning with pGL4.74 (renilla) and pGL4.10 (firefly) vectors and luciferase assays were carried out as described previously [25]. All cloning primers and vector maps are listed in Table S1. Transfection was performed using JetPei (Polyplus, New York, NY, USA) transfection reagent according to the manufacturer’s instruction. An empty vector served as a negative control for gene overexpression. Cells were harvested 72 h post transfection. Primer sequences are listed in Table S1.

### 2.6. RNA Isolation, cDNA Synthesis and qPCR

RNA and miRNA were isolated using the RNeasy Mini Kit and miRNeasy Mini Kit (both Qiagen, Hilden, Germany), respectively, according to the manufacturer’s protocol. cDNA synthesis was performed using the RevertAid First Strand cDNA Synthesis Kit (Thermo Fisher Scientific, Waltham, MA, USA) according to the supplier’s instructions. For miRNA quantitation, target miRNA and a control miRNA (RNU6B) were transcribed using the TaqMan MicroRNA Reverse Transcription Kit (Thermo Fisher Scientific) and primers from TaqMan MicroRNA Assays (Thermo Fisher Scientific). Quantitative PCRs were undertaken as described previously [24] using the LightCycler 480 Instrument II (Roche, Mannheim, Germany). Primer sequences are listed in Table S1.

### 2.7. Expression Profiling

Expression profiling using Illumina HT-12 Bead Chips (Illumina, San Diego, CA, USA) was accomplished as described previously [24] and deposited under GSE78032.

### 2.8. Immunofluorescence

Cells were grown in 8-well µ-plates (IBIDI, Gräfelfing, Germany) and further processed as described previously [26]. The primary antibodies are listed in Table S1.

### 2.9. Co-Immunoprecipitation (Co-IP)

Harvested cells were incubated rotating at 4 °C for 30 min and sheared protein concentration was determined. Lysate was diluted to 1 µg/µL with low detergent RIPA (150 mM NaCl (Thermo Fisher Scientific), 50 mM Tris-HCl (Sigma), 1 mM EDTA (Sigma), 1 × cOmplete Mini (Merck, Darmstadt, Germany), 1× PhosSTOP (Merck)). Magnetic antibody-crosslinked beads (ChIP-Grade Protein G Magnetic Beads; New England Biolabs, Frankfurt, Germany) were washed twice in PBS and 500 µL of diluted protein lysate were incubated with 30 µL magnetic beads per IP overnight, gently shaking at 4 °C. Elution was performed with 30 µL of 0.5 M glycine (pH: 3). Protein eluates were either submitted to the DKFZ Genomics and Proteomics Core Facility for mass spectrometry (MS) and peptide identification or used for Western blotting to verify MS predicted interaction partners.

### 2.10. Chromatin Immunoprecipitation (ChIP)

ChIP was performed as described previously [25]. Briefly, 1 × 10^6^ formaldehyde-fixed LNCaP cells were sheared and incubated with 2 μg antibody (Table S1). Immunoprecipitation was performed by adding 30 μL prewashed Protein G Magnetic Beads (Cell Signaling Technology). The bead-separated eluted chromatin was purified using the High Pure PCR Template Preparation Kit (Roche) according to the supplier’s instructions, eluted in 50 µL elution buffer and further diluted with 100 µL ultrapure H_2_O.

### 2.11. Proliferation Assay

Proliferation assays were carried out in 96-well culture vessels with 2 × 10^4^ cells in 93 µL medium per well. For every aimed time point of WST-1 analysis one 96-well plate was prepared. Per well, 7 µL siRNA transfectant were added. At the day of analysis 10 µL WST-1 reagent (Roche) were added and plates incubated for 2 h, 3 h, and 4 h, respectively. The absorbance was measured using the Tecan infinite M200 microplate reader (Tecan, Männedorf, Switzerland) at 450 nm.

### 2.12. Colony Formation Assay

LNCaP cells were subseeded in a 6-well culture vessel 72 h post-treatment at 2000 cells per well and incubated for 10 days. Medium was removed, colonies were gently washed using 2 mL PBS per well and cells were fixed with 100% methanol. After staining with 0.005% crystal violet solution pictures were taken using the ChemiDoc XRS+ System (Bio-Rad Laboratories, Feldkirchen, Germany), Coomassie mode, and colony count was performed using the Software OpenCFU [27].

### 2.13. Migration Assay

Migration assays were carried out in duplicates using 24-well transwell chambers (Thincert cell culture insert (Greiner Bio-One, Frickenhausen, Germany)) with 8 μm pore size. Every transwell chamber was filled with 1 × 10^5^ LNCaP cells in 250 µL FBS-free medium and 750 µL full growth medium in the bottom well. Cells were incubated for 48 h at 37 °C before fixation in 1 mL 70% ethanol for 15 min and staining using 0.02% crystal violet. Membranes were imaged using the Cell Observer microscope (Zeiss, Jena, Germany) and cells in four equally sized fields were selected to count migrated cells in both biological replicates.

### 2.14. 3D Invasion Assay

In a 96-well culture vessel, 40 µL matrigel (Corning Matrigel Matrix, phenol red free (VWR International GmbH, Darmstadt, Germany)): medium (1:1) suspension were added to the wells and allowed to solidify for 1 h at 37 °C. Next, 2000 cells in 45 µL full growth medium containing 10 mM Batimastat and 10 mM MK-0752 (Biomol, Germany, Hamburg) or DMSO (AppliChem, Germany, Darmstadt) were mixed with 15 µL Matrigel, added to the wells and allowed to solidify for 4 h at 37 °C. As final step, 180 µL growth medium containing 10 mM Batimastat and 10 mM MK-0752 or DMSO were layered on top. The cells were incubated for 7 days and imaged using the Cell Observer microscope. For quantification, compact and invasive spheroids in random equally sized frames were counted throughout all replicates.

### 2.15. Cell Cycle Assay

Cells were fixed in 66 % ice cold ethanol for 2 h, gently resuspended, centrifuged (500× *g* for 5 min) and washed in 1 mL ice cold PBS. Afterwards the cells were stained in 200 µL 1× propidium iodide supplemented with RNase staining solution (Thermo Fisher Scientific). Cells were incubated for 30 min in the dark and analyzed with the FACS Canto (BD Biosciences, Franklin Lakes, NJ, USA).

### 2.16. Microscopy

Brightfield and fluorescence microscopy pictures were imaged using the Cell Observer microscope (Zeiss, Jena, Germany).

### 2.17. Tissue Microarrays

Tissue microarrays (TMAs) were executed (on cohort I) as described previously [28]. For the TMAs, 165 patients were enrolled between 1990 and 2010. Per patient, one to four tissue biopsies were analyzed for HES1 and ERG expression (Table S2). The cohort consists of mostly high-grade PCa, classified by an experienced pathologist. Of 683 TMA cores, 150 were excluded from further analysis due to insufficient sample quality.

### 2.18. Statistical Analysis

Statistical analysis was performed on pre-processed TCGA mRNA-seq data using a Bonferroni corrected two-tailed *t*-test. For HumanHT-12 v4 expression BeadChip (Illumina) outlier removal the M-estimator method was applied; the remaining data points were quantile normalized. Significance was tested using a two-tailed *t*-test. *p*-values for the differential expression analysis were Benjamini-Hochberg corrected. The cell culture experiments were conducted in three biological and, where indicated, in technical triplicates. Data are presented as mean ± standard error (SE). Statistical testing was performed using a two tailed *t*-test to reject or accept the null hypothesis. For qPCR results, fold changes were log2 normalized prior to *t*-testing, to obtain a normal distribution.

## 3. Results

### 3.1. The miR-449 Family Is Associated with Enhanced NOTCH Signaling in T2E+ PCa

**Figure 1:**
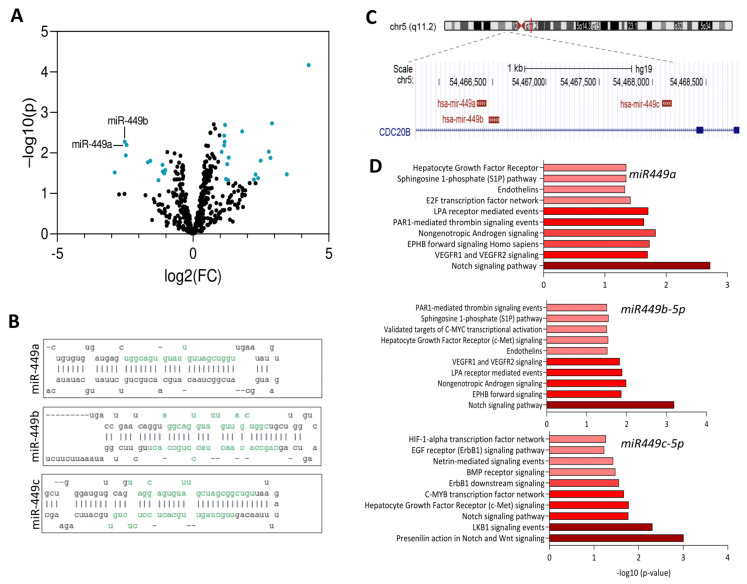
The miR-449 family is associated with enhanced NOTCH signaling in T2E+ PCa. (**A**) Volcano plot of miRNAs [22] with log2(fold changes) annotated on the *x*-axis and −log10(*p*-values) annotated on the *y*-axis. (**B**) Sequences and structures of miR-499a, -b, and -c (mirbase.org). (**C**) Location of miR449a, -b, and -c on chromosome 5 (genome.ucsc.edu). (**D**) Bargraph of signaling pathways affected by predicted target genes of miR-449a, miR-449b-5p and miR-449c-5p.

In order to identify global genomic and epigenomic alterations correlating with T2E, we first performed analyses of the TCGA-PRAD data grouped according to their ERG expression levels (Figure S1). We found that the presence of the T2E gene fusion correlates not only with a specific global mRNA expression pattern, but also with T2E-specific DNA methylation and H3K27ac profiles [4]. In addition, T2E expression correlates—to a lower extent—with an intrinsic miRNA expression pattern (Figure S2). Next, we analyzed the top miRNAs repressed in T2E+ versus T2E– PCa from our previous study based on derived fold changes and p-values out of 522 miRNAs from 47 tumor specimens (GSE29079) [22]. *miR-449a* and *miR-449b* belonged to the most significantly repressed transcripts in the evaluated cohort (Figure 1A). *miR-449a* and *miR-449b* are part of a cluster with *miR-449c*. Clusters of miRNAs frequently share upstream regulatory elements and are therefore often coexpressed [29]. The *miR-449* precursor miRNAs are located in genomic proximity to each other on chromosome 5q11 and exhibit high sequence homologies (Figure 1B, C). The significant repression of *miR-449a* in T2E+ versus T2E− PCa in the TCGA-PRAD cohort (*p* = 0.02) and a previous study of miRNAs in twelve PCa tissue specimens corroborated this result [30]. Additionally, these findings were supported by small RNA-seq data using the ICGC Early Onset Prostate Cancer cohort (ICGC-EOPC; unpublished data) [23].

Bioinformatic target gene prediction as well as pathway analysis of the predicted target genes [31] revealed repression of NOTCH signaling by the *miR-449* cluster. In detail, target prediction of *miR-449a* resulted in 891 target genes. The pathway most significantly associated with deregulation of those genes was NOTCH signaling (adj. *p* = 0.002). Similarly, target prediction of *miR-449b-5p* revealed 801 target genes, also presenting NOTCH as the most significantly enriched pathway (adj. *p* > 6.48 × 10^−4^), whereas for *miR-449c-5p* 566 target genes were predicted, resulting in activation of “Presenilin action in NOTCH and WNT signaling” (adj. *p* > 9.88 × 10^−4^) (Figure 1D; Table S3).

**Figure 2:**
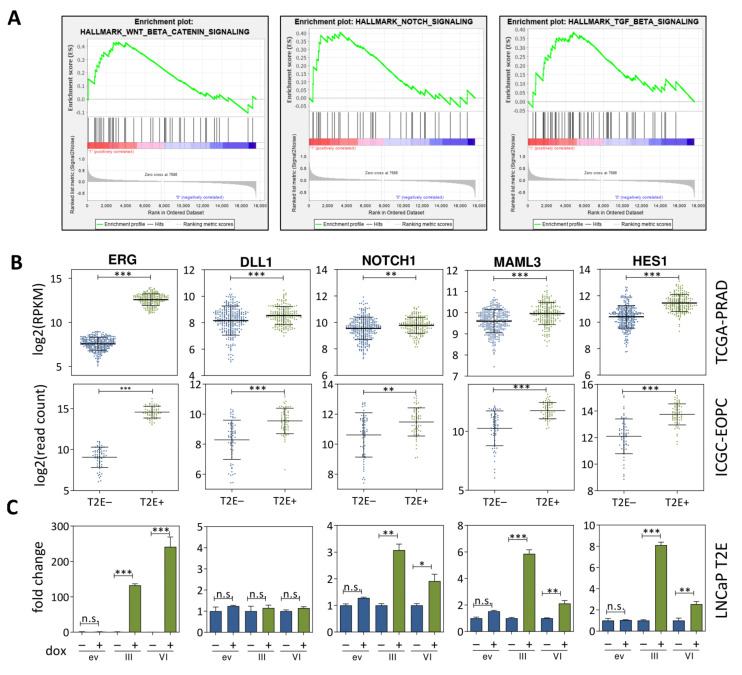
NOTCH signaling is enhanced in T2E+ tissue specimens and cell lines. (**A**) Gene set enrichment analysis plots identifying WNT/β-catenin, NOTCH and TGF-β signaling as positively enriched sets in T2E+ compared to T2E− patient samples. (**B**) Expression of *ERG* and four *NOTCH* signaling genes separated according to T2E fusion (T2E−, T2E+) status estimated from *ERG* expression of TCGA-PRAD and ICGC-EOPC data. (**C**) Fold changes of mRNA expression measured via qPCR of NOTCH signaling genes in LNCaP T2E cells. Empty vector (ev), T2E fusion variant III (III), and T2E fusion variant VI (VI) were treated with PBS (−) or doxycycline (+). Bars representing cells with *ERG* overexpression are displayed in green and control bars in blue (n.s. not significant, * *p* ≤ 0.05; ** *p* ≤ 0.01; *** *p* ≤ 0.001).

Since miRNAs silence mRNAs, we investigated if the downregulation of miRNAs in T2E+ PCa contributed to an increased expression of genes and gene sets. Gene Set Enrichment Analysis (GSEA) of the TCGA-PRAD mRNA-seq data identified an enrichment of 15 out of 50 hallmark gene sets (MsigDB) (Table S4). Among the top enriched hallmark gene sets (Figure S3) were WNT/β-catenin, NOTCH, and TGF-β signaling (Figure 2A).

TGF-β and WNT signaling pathways have previously been described to play a role in T2E+ PCa by our group and others [22,24,32]; first results on NOTCH signaling in T2E+ were published recently [9]. Significant upregulation of prominent NOTCH pathway genes, e.g., *DLL1*, *NOTCH1*, *MAML3*, and *HES1*, was observed in T2E-positive tumors in both, the TCGA-PRAD and ICGC-EOPC data cohorts (Figure 2B). To investigate direct consequences of the T2E fusion in vitro, we previously established a Tet-On system for doxycycline (dox)-inducible gene expression of the T2E gene fusion variants T2E III (T1:E4) and T2E VI (T1:E5) in LNCaP cells (LNCaP T2E) (Figure S4A), and investigated the influence of ERG overexpression in these LNCaP T2E cells using microarray gene expression profiling [24]. The most significantly deregulated pathway according to Biocarta 2016 analysis [33] was Vascular Endothelial Growth Factor (VEGF) and Angiogenesis (*p* = 6.46 × 10^−5^) (Table S5). This is of particular interest since ERG is a prominent transcription factor in endothelial cells, where NOTCH signaling is known to enhance angiogenesis [11,34,35]. Furthermore, *ERG* overexpression for 72 h led to a significant upregulation of *NOTCH1*, *MAML3* and *HES1* mRNA and NOTCH1 and HES1 protein in LNCaP T2E cells (Figure 2C, Figure S4B). The activation of NOTCH signaling requires the sequential cleavage of NOTCH1 by α-secretases such as ADAM10 and ADAM17 followed by cleavage through γ-secretase [36]. In LNCaP cells, the combinatorial knockdown of *ADAM10* and *ADAM17* led to significantly reduced *NOTCH1* and *HES1* expression (Figure S5). This indicated a functional NOTCH signaling pathway in those cells.

**Figure 3:**
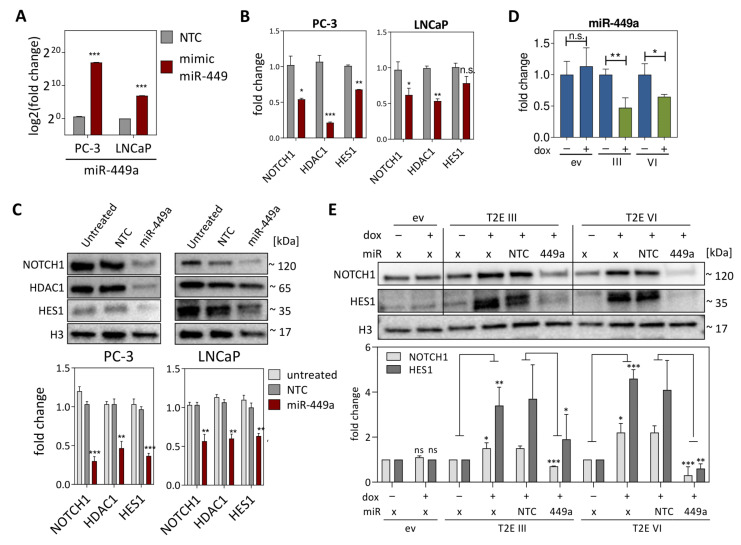
Gene regulation by *miR-449a* in PCa cell lines. (**A**) Quantitation of *miR-449a* mimic levels after transfection into PC-3 and LNCaP cells. (**B**,**C**) mRNA and protein levels of NOTCH1, HDAC1 and HES1 in PC-3 and LNCaP cells 72 h after *miR-449a* mimic transfection compared to non-target control (NTC). Histone H3 served as internal control. (**D**) *miR-449a* expression in LNCaP T2E cells without (blue) and with (green) induction of ERG with doxycycline (+). PBS treatment (−) served as control. (**E**) Western blot and protein quantification of NOTCH1 and HES1 in LNCaP T2E ev, III and VI cells, respectively, transfected with no/control mimic/*miR-449a* mimic (x/NTC/449a) for 48 h, followed by repeated miRNA mimic transfection without/with (−/+) PBS/doxycycline treatment for 72 h (n.s. not significant, * *p* ≤ 0.05; ** *p* ≤ 0.01; *** *p* ≤ 0.001).

Since *miR-449a* has already been described to support tumor suppressive properties in different cancer entities [37,38,39], and to repress NOTCH signaling [38,40,41,42,43], we investigated the influence of *miR‑449a* mimic transfection on predicted target genes associated with NOTCH signaling in LNCaP and PC-3 cells (Figure 3A). Decreased mRNA and protein levels were observed for NOTCH1, HDAC1 and HES1 (Figure 3B,C). Next, *miR-449a* expression levels were measured in the LNCaP T2E model. In line with PCa patient-derived data, induction of T2E led to reduced *miR-449a* expression to 0.47 − (T2E III dox) and 0.64-fold (T2E VI dox), respectively (Figure 3D). To verify the influence of *miR-449a* on NOTCH signaling genes in vitro, LNCaP T2E cells overexpressing *ERG* were monitored for *NOTCH1* and *HES1* expression, with and without *miR-449a* mimic transfection. Induction of *ERG* led to increased NOTCH1 and HES1 protein levels, which in turn could be diminished by cotransfection with *miR-449a* mimics (Figure 3E). Vice versa, transfection of LNCaP cells with *miR-449* inhibitor, led to increased NOTCH1 and HDAC1 protein levels (Figure S6).

Taken together, we found several miRNAs belonging to the *miR-449* cluster to be repressed in T2E+ tumors. Computational analysis revealed that repression of this miRNA cluster is highly associated with enhanced NOTCH signaling. Key genes of the NOTCH signaling pathway are significantly increased in T2E+ tumors of the TCGA-PRAD and ICGC-EOPC mRNA-seq datasets. Using a doxycycline-inducible LNCaP T2E model we confirmed the repression of *miR-449a* and enhancement of NOTCH signaling upon induction of the ERG fusion transcript in vitro. This effect was reversed by transfection of *miR-449a* mimics.

### 3.2. NOTCH Signaling Promotes Oncogenic Properties of PCa Cells

**Figure 4:**
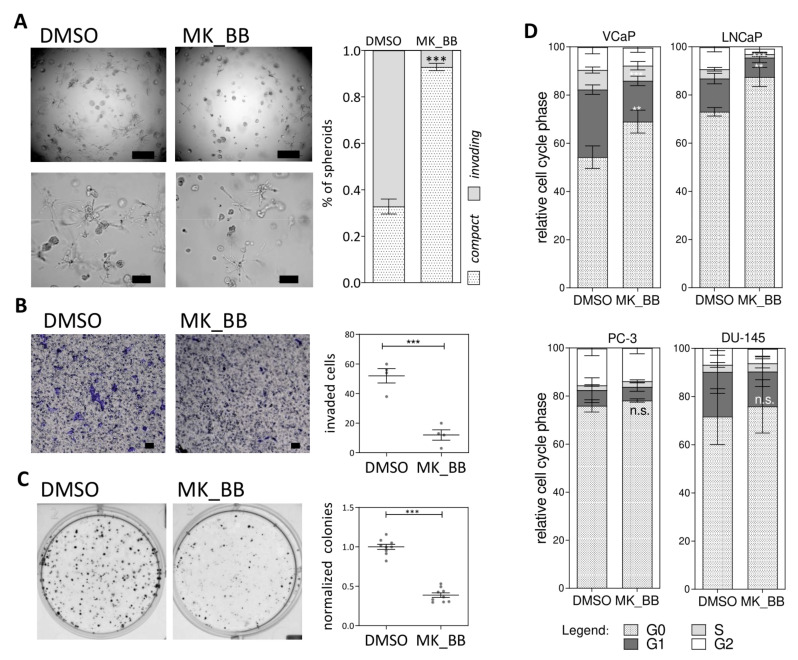
NOTCH signaling enhances PCa cell migration and invasion in vitro. (**A**) 3D invasion assay of PC-3 spheroids. PC-3 cell spheroids in DMSO control, and MK-0752 (MK) Batimastat (BB)-treated conditions. Quantitation of invaded spheroids was performed in three replicates in three randomly selected fields. Scale bars indicate 500 µm (**top**) or 200 µm (**bottom**) of length. Stacked bar plots visualize quantitated compact and invading PC-3 spheroids. (**B**) LNCaP migration assay with LNCaP cells. The scale bar indicates 200 µm of length. Quantitation of migrated cells was performed after crystal violet staining in four randomly selected fields. (**C**) LNCaP colony formation assay. The number of colonies was determined for three biological and technical replicates using the OpenCFU software. (**D**) Cell cycle assay of VCaP, LNCaP, PC-3 and DU-145 cell lines upon NOTCH inhibition (n.s. not significant, ** *p* ≤ 0.01; *** *p* ≤ 0.001).

To address if NOTCH signaling influences the aggressiveness of PCa cells in vitro, cellular assays upon pharmacological NOTCH inhibition were performed: we inhibited γ-secretase with MK-0752 (MK) and metalloproteinases with Batimastat (BB) and first applied a 3D spheroid invasion assay to investigate invasive and clonogenic capabilities of PCa cells. NOTCH inhibition significantly reduced the number of invading spheroids (Figure 4A). Subsequently, a significant reduction of migrated cells and colony formation capacities was observed upon inhibiting NOTCH signaling in 2D cultures (Figure 4B,C). Since NOTCH signaling has been described to mediate G1/S cell cycle progression [44] we also performed cell cycle assays upon MK_BB treatment in VCaP, LNCaP, PC-3 and DU-145 cell lines. Pharmacological inhibition of NOTCH with MK_BB resulted in a G0 cell cycle arrest in all cell lines; statistical significance for the cell cycle arrest was observed in VCaP and LNCaP cells (Figure 4D). The results of the invasion, migration and clonogenic assays upon NOTCH inhibition strongly suggested that NOTCH signaling promotes tumorigenic properties PCa tumor cells in vitro.

### 3.3. The HDAC1-KDM1A Interaction Inhibits the HES1 Repressor HES6

**Figure 5:**
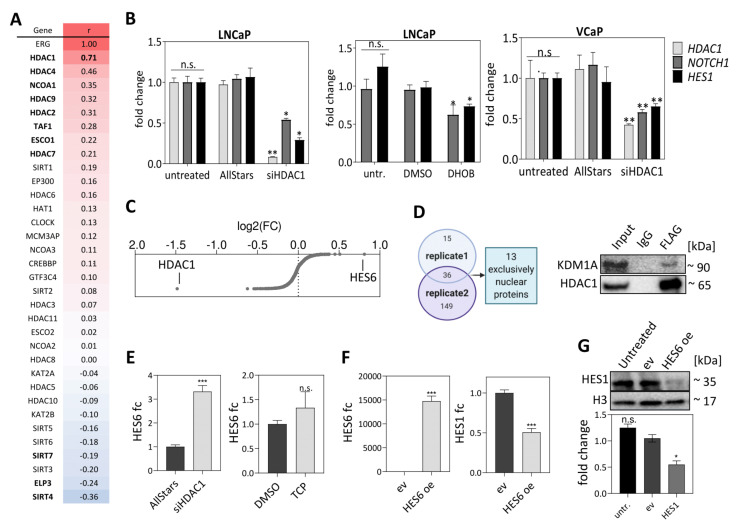
HDAC1 enhances NOTCH signaling in PCa cell lines. (**A**) *HDAC1* is the top *ERG*-correlated epigenetic modifier in the TCGA-PRAD data cohort (Spearman correlation coefficients; positive: red; negative: blue). (**B**) siRNA-mediated knockdown of *HDAC1* in LNCaP and VCaP cells, and treatment with the *HDAC1* inhibitor DHOB in LNCaP cells. (**C**) Fold changes resulting from expression profiling of LNCaP cells upon *HDAC1* knockdown. (**D**) Co-immunoprecipitation of FLAG-tagged HDAC1 followed by mass spectrometry resulting in a set of 13 nuclear proteins from two replicates, and Western blot of HDAC1-KDM1A interaction verification. (**E**) *HES6* expression levels upon *HDAC1* knockdown or KDM1A inhibition by trancyclopromine (TCP) in LNCaP cells (fc: fold change). (**F**) Overexpression of *HES6* (**left**) leads to reduced *HES1* expression (**right**) in LNCaP cells. (**G**) HES1 protein expression levels are reduced upon *HES6* overexpression in LNCaP cells. (n.s. not significant, * *p* ≤ 0.05; *** *p* ≤ 0.001).

The expression of the epigenetic modifier gene *HDAC1*, which is part of a NOTCH signaling co-repressor complex [45], is not only repressed by *miR-449a* (Figure 3B,C), but also highly correlated to *ERG* in the ICGC-EOPC (r = 0.72, *p* < 2 × 10^−16^) and TCGA-PRAD data cohorts (r = 0.71, *p* < 2 × 10^−16^) (Figure 5A and Figure S7). Knockdown of *HDAC1* in LNCaP and VCaP cells reduced the expression of key genes of the NOTCH signaling pathway, such as *NOTCH1* or *HES1*. Similar results were also obtained in LNCaP cells using 10 µM of the HDAC1-inhibitor 4-(dimethylamino)-N-[6-(hydroxyamino)-6-oxohexyl]-benza- mide (DHOB) (Figure 5B). To evaluate whether *HDAC1* plays a distinct role in NOTCH signaling gene regulation, LNCaP cells were treated with the histone deacetylase inhibitors abexinostat (PCI-24781) or entinostat (MS-275), which led to concentration-dependent upregulation of the NOTCH signaling genes *DLL1*, *NOTCH1* and *HES1* (Figure S8A). This upregulation was accompanied by enrichment of H3K27ac marks in the corresponding gene promoter regions (Figure S8B), suggesting that increased expression of multiple HDACs leads to decreased H3K27ac marks and subsequent repression of target genes. However, global gene expression profiling upon *HDAC1* knockdown in LNCaP cells revealed that 50% of the significantly deregulated transcript variants were upregulated (n = 140, log2(FC) > |0.5|) (Figure 5C), indicating that HDAC1 cofactors might be modulating its activity. Therefore, we performed FLAG-tagged HDAC1 immunoprecipitation followed by mass spectrometry in LNCaP cells and identified 36 HDAC1-interacting proteins (Table S6). Thirteen of these proteins could be clearly assigned to the nuclear compartment. Besides KDM1A, known histone proteins such as HIST1H4A, and chaperones such as HSP90AB1, were enriched in the pulldown, as expected. Interaction of HDAC1 with the lysine demethylase KDM1A, both proteins of the CoREST complex, was verified by immunoprecipitation followed by Western blot analysis (Figure 5D).

The expression profiles of LNCaP cells upon *HDAC1* knockdown also revealed *HES6* to be the most highly upregulated (FC: 1.8) transcription factor (Figure 5C), which was validated by qPCR (FC: 3.3). Similarly, inhibiting *KDM1A* using 50 µM tranylcypromine (TCP) resulted in 1.3-fold increased *HES6* mRNA levels (Figure 5E). Since HES6 is known to be a repressor of *HES1* [46,47], we overexpressed *HES6* in LNCaP cells and found that it represses *HES1* on mRNA (Figure 5F) and protein (Figure 5G) levels by 50% each. These data suggest that the direct interaction of HDAC1 and KDM1A leads to repression of *HES6*, which in turn leads to enhanced *HES1* expression and positive regulation of NOTCH signaling in PCa cells.

### 3.4. ERG Can Directly Induce NOTCH1 Transcription

**Figure 6:**
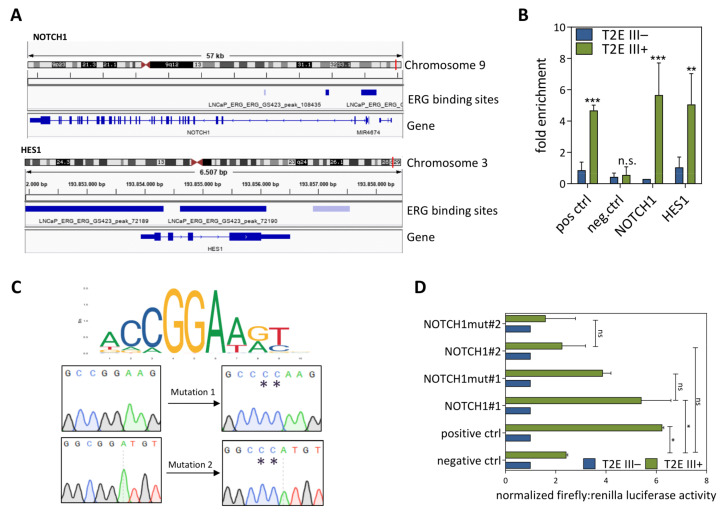
The transcription factor ERG binds to *NOTCH1* and *HES1* promoter regions. (**A**) Chromosomal locations of *NOTCH1* and *HES1* and ERG ChIP-seq binding sites displayed in the IGV browser (ChIP-Seq data from [48]). (**B**) ERG ChIP-qPCR of *NOTCH1* and *HES1* promoter regions in LNCaP T2E III cells induced with PBS (−) or doxycycline (+). (**C**) Mutation insertion in *NOTCH1* promoter regions. Jaspar [49] annotated ERG binding site sequence logo and Sanger sequencing results of wildtype and mutated NOTCH1#1 (**top**) and NOTCH1#2 (**bottom**) ERG binding sites. (**D**) Normalized firefly:renilla luciferase activity of the different *NOTCH1*, mutated *NOTCH1*, and control promoter regions in control (blue) and doxycycline (+, green) treated LNCaP T2E III cells (n.s. not significant, * *p* ≤ 0.05; ** *p* ≤ 0.01; *** *p* ≤ 0.001).

We hypothesized that deregulation of *miR-449a* was not the only mechanism enhancing NOTCH signaling, since signaling pathway regulation is frequently multifactorial. To this end, we analyzed the publicly available LNCaP-derived ERG ChIP-seq data for ERG binding sites in promoter regions of NOTCH signaling genes. The most abundant peaks were found in the promoter regions of the *NOTCH1* and *HES1* genes (Figure 6A). To verify ERG binding at these genomic positions, we performed ERG ChIP-qPCR and found a significant enrichment for ERG-chromatin interaction in the *NOTCH1* and *HES1* promoters (Figure 6B). To verify ERG binding, a luciferase reporter assay was designed to characterize two ERG binding sites (#1, #2) with the highest relative scores within the *NOTCH1* promoter region. ERG-mutated binding sites were used as negative controls, and sequence verified in the two constructs (Figure 6C). The promoter region of *HLA-DMB*, the most highly *ERG*-correlated gene in the TCGA-PRAD cohort (r = 0.75, *p* < 2.2 × 10^−16^), served as a positive control. A significant increase of luminescence ratios between doxycycline-induced LNCaP T2E III of negative versus positive firefly vector control transfections was observed (*p* < 0.02). In addition, a significant increase of luminescence between negative control and NOTCH1#1 in T2E III+ cells (*p* < 0.045) in comparison to NOTCH1mut#1 was found. LNCaP T2E III+ cells transfected with promoter region NOTCH1#2 firefly vector showed no significant increase in luminescence ratio compared to the negative control (Figure 6D).

### 3.5. HES1 Represses the Expression of the Cell Cycle Inhibitor p21

**Figure 7:**
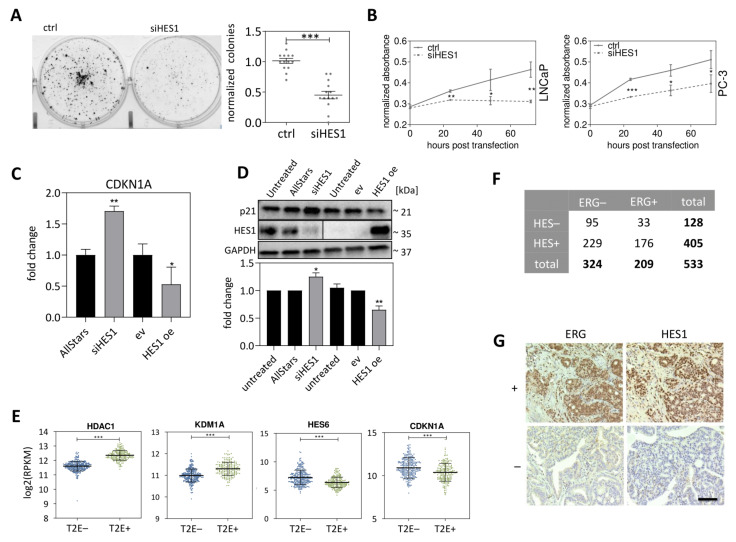
Consequences of *HES1* knockdown and overexpression in T2E+ PCa. (**A**,**B**) Colony formation and proliferation assays upon *HES1* knockdown. Representative images of (**A**) LNCaP colony formation assays and (**B**) LNCaP and PC-3 WST-1 assays in scrambled siRNA (ctrl), and *HES1* siRNA (siHES1) treated conditions. (**C**) *CDKN1A* mRNA expression upon siRNA-mediated *HES1* knockdown and transient *HES1* overexpression (HES1oe). (**D**) Western blot of HES1 and p21 after *HES1* expression perturbation (Blot is derived from two different, parallel processed membranes due to HES1-GAPDH signal interference). (**E**) *HDAC1*, *KDM1A*, *HES6* and *CDKN1A* expression in PCa tissue specimens from the TCGA-PRAD data cohort (* *p* ≤ 0.05; ** *p* ≤ 0.01; *** *p* ≤ 0.001). (**F**) Contingency table of ERG/HES1 tissue microarray data. (**G**) Representative images of positive and negative staining of PCa tissue microarray for ERG and HES1, respectively (scale bar indicates 150 µm).

As *HES1* is the main target gene of NOTCH signaling affected by the T2E gene fusion in PCa, phenotypical effects of *HES1* knockdown were evaluated using in vitro assays. Similar to the combined γ-secretase and metalloprotease inhibition by MK_BB, *HES1* knockdown resulted in significantly reduced clonogenic capacity of LNCaP cells (Figure 7A). In agreement with the cell cycle arrest observed upon NOTCH inhibition (Figure 3A), siRNA-mediated *HES1* knockdown led to significantly decreased cell proliferation (Figure 7B). These phenotypical changes suggested that the observed cell cycle arrest and reduced clonogenic capacities in PCa cell lines upon combined γ-secretase and metalloprotease inhibition were mediated by *HES1*. One of the most promising targets of the transcriptional repressor *HES1* is *CDKN1A*/p21 [50]. *CDKN1A* mRNA expression levels and p21 protein expression levels were quantified upon *HES1* expression perturbation. Knockdown of *HES1* resulted in 1.7-fold upregulation of *CDKN1A* mRNA (Figure 7C) and 1.4-fold upregulation of p21 (Figure 7D), whereas overexpression of *HES1* led to a 2-fold downregulation of *CDKN1A* on mRNA (Figure 7C) and a 1.7-fold downregulation of p21 on protein levels (Figure 7D). These in vitro data were supported by the TCGA-PRAD cohort, where T2E+ PCa tissue specimens exhibited significantly decreased *CDKN1A* and *HES6* expression levels compared to T2E-PCa, while *HDAC1* and *KDM1A* were increased (Figure 7E).

### 3.6. HES1 Protein Expression Is Elevated in T2E+ PCa Tissue Specimens

To investigate the relevance of our finding in PCa tumors, we determined the correlation between ERG and HES1 expression in patient samples. To this end, ERG and HES1 protein levels in T2E+ versus T2E– PCa were analyzed using tissue microarrays (TMAs) [28] encompassing one to eight tissue core biopsies from 165 high grade PCa patients. Of 683 biopsy cores, 150 were excluded from further analysis due to insufficient sample quality. From the remaining 533 tissue cores, 209 exhibited positive staining for ERG, and 324 were ERG-negative. With 176 HES1-positive detections on the ERG-positive cores and 229 HES1-positive detections on the 324 ERG-negative cores, the hypothesis that ERG-positive tumors express higher levels of HES1 compared to ERG-negative tumors was supported by the statistical analysis (*p* > 0.001) (Figure 7F). Representative images of the tissue microarray cores for ERG and HES1 staining are depicted in Figure 7G.

## 4. Discussion

**Figure 8:**
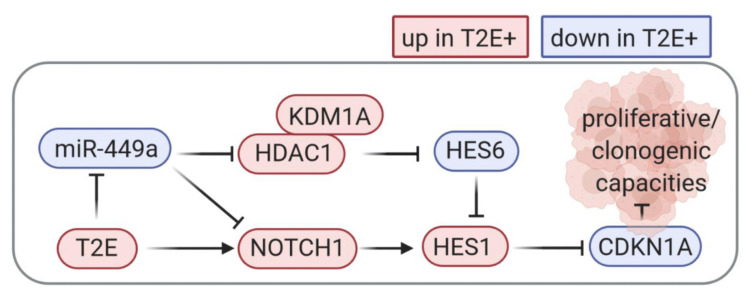
Proposed regulatory network of NOTCH signaling in T2E+ PCa. Genes with blue colored background are downregulated, and genes with red colored background are upregulated, respectively, in LNCaP T2E and TCGA-PRAD derived data. Arrows and stop bars define activation and inhibition.

This study reveals novel regulatory mechanisms leading to enhanced NOTCH signaling in T2E+ PCa. We identified the *miR-449* cluster to be strongly repressed in fusion-positive tumors. *miR-449a* was shown to repress NOTCH signaling in vitro, a finding which correlated with clinical data. In addition, we also found *HDAC1* to promote NOTCH signaling indirectly by inhibiting *HES6*, a transcriptional repressor of *HES1*. Furthermore, ERG binds to the *NOTCH1* promoter region and induces its transcription. Finally, we were able to confirm that NOTCH signaling increases invasiveness and clonogenic capabilities of prostate cancer cells. The network of interactions is illustrated in Figure 8.

Among the top repressed miRNA transcripts in T2E+ PCa are miRNAs belonging to the *miR-449* cluster. *miR-449a* is frequently repressed in various tumor entities, thereby promoting oncogenic properties [51,52]. Regulation of the *miR-449* cluster is associated with alterations in H3K27ac marks, which can also be mediated by inhibition of histone deacetylation [53,54]. In publicly available PCa cell line-derived ChIP-seq data we identified a HDAC1 binding-site in the CpG island located upstream of the *miR-449* cluster, which is associated to a 152 kB upstream enhancer [55,56], suggesting a feed forward loop in the *HDAC1-miR-449a* regulation. Involvement of *miR-449a* in NOTCH signaling regulation has been reported for vertebrate cilia formation [57] and cell fate determination [58]. Loss of *miR-449a* enhances *HDAC1* expression leading to reduced proliferation of PC-3 cells [51], or to enhanced PrLZ (prostate leucine zipper) expression, which promotes PCa progression and metastasis formation [40]. Predicted target genes for *miR-449a*, *miR-449b-5p* and *miR-449c-5p* were clearly associated with NOTCH signaling and experimentally verified in this work. For *HDAC1* and *NOTCH1*, a direct regulation via *miR-449a* binding to the 3′UTR was experimentally verified via luciferase assays in previous publications [43,59]. Since *HES1* is not a predicted *miR-449a* target gene, an indirect regulation via *NOTCH1* is likely.

NOTCH mediates cell-cell communication via ligand-receptor interactions between signal-sending and signal-receiving cells. The 3D invasion assay was performed since increasing cell-cell surface contact leads to elevated NOTCH signaling [60]. In different cancers, NOTCH signaling exhibits tumor repressive or oncogenic properties [61]. We observed that NOTCH signaling inhibition or *HES1* knockdown in PCa cells reduced their oncogenic properties and, therefore, indicate an oncogenic role of NOTCH signaling in PCa.

HDAC1 is a transcriptional repressor of NOTCH signaling, which acts as a co-repressor of NOTCH signaling target genes [62]. In contrast, in PCa cells, we identified HDAC1 as a positive regulator of *NOTCH1* and *HES1*. Further characterization of HDAC1 in the NOTCH signaling pathway was of particular interest since *HDAC1* was highly correlated to ERG expression in clinical samples on both mRNA and protein levels [63]. Knockdown of *HDAC1* in VCaP and LNCaP cells reduced the expression of *NOTCH1* and *HES1*. Since H3K27ac is an activating histone mark, which is associated with active promoters [64], knockdown of *HDAC1* would result in genome-wide retention of H3K27ac marks and therefore contribute to enhanced gene expression. However, in the expression profiling data of LNCaP cells upon *HDAC1* knockdown, 50% of the deregulated genes were repressed and therefore a bias towards increased gene expression was not observed. HDAC1 can also enhance gene expression by functioning as a transcriptional co-activator [65]. Using co-immunoprecipitation of FLAG-tagged HDAC1, no transcription factor was identified among the 36 proteins interacting with HDAC1. However, the histone demethylase KDM1A, a known interaction partner of HDAC1 [66], was highly enriched. HDAC1 and KDM1A are components of the CoREST complex, which is associated with epigenetic silencing of genes [67]. Thus, the upregulation of transcriptional repressors upon *HDAC1* knockdown could also facilitate the repression of several genes. To this end, *HES6* exhibited the strongest upregulation (2.6-fold) in the expression profiling experiment after *HDAC1* knockdown. HES6 is a transcriptional repressor of *HES1* [46], which was confirmed by overexpression of *HES6* in LNCaP cells, followed by subsequent downregulation of *HES1* RNA and protein. Published data suggest that silencing of *KDM1A* in the LNCaP-derived C42B cell line also upregulated *HES6* by 1.9-fold (GSE61630) [68].

ERG is not the only driver of NOTCH signaling in PCa, and NOTCH signaling is also active in T2E– tumors. Basal canonical NOTCH signaling is described to be active in all multicellular organisms and all human tissues to regulate angiogenesis, vascularization and tissue homeostasis. Thereby the NOTCH receptor, produced in the endoplasmic reticulum, is presented on a juxtaposed cell. After interaction with its ligand, the S2 and S3 cleaved intracellular NOTCH domain (NICD) is translocated to the nucleus to form an activator complex and promote target gene expression [18]. However, the T2E fusion and therefore ERG expression obviously enhances NOTCH expression in PCa cells.

In summary, our data unraveled a novel molecular network driving NOTCH signaling in T2E+ PCa cells (Figure 8). We show that, on the one hand, ERG directly activates *NOTCH1* and *HES1* expression by interacting with their promoter regions. On the other hand, NOTCH is activated by downregulation of *miR-449a* in ERG overexpressing cells. Experimental NOTCH pathway inhibition as well as *HES1* knockdown reduced oncogenic capacities in vitro, suggesting that the NOTCH pathway triggers oncogenic rather than tumor suppressive processes in T2E+ PCa. The enhanced expression of HES1 correlates with ERG expression in tissue samples from PCa patients.

## Data Availability

Publicly available datasets used in this study can be found at www.ncbi.nlm.nih.gov/geo/; prostate cancer miRNA profiling: GSE29079; gene expression LNCaP LSD1 (KDM1A) knockdown: GSE61630; gene expression LNCaP HES6 overexpression: GSE36526; gene expression LNCaP ERG overexpression: GSE78032; gene expression LNCaP HDAC1 knockdown: GSE159839; LNCaP ERG ChIP-Seq: GSE110657. Mass spectrometry data is contained within the Supplementary Materials (Table S5).

## Supplementary Materials

The following are available online at https://www.mdpi.com/2072-6694/13/5/964/s1. Figure S1: Sequencing cohort ERG sample separation; Figure S2: T2E+ specific profiles in the TCGA-PRAD cohort; Figure S3: Gene set enrichment analysis of TCGA-PRAD samples; Figure S4: Tet-On doxycycline-inducible empty vector, III and T2E VI in LNCaP T2E cells; Figure S5: SiRNA mediated ADAM10 and/or ADAM17 knockdown in LNCaP cells; Figure S6: *miR-449* inhibition in LNCaP cells; Figure S7: *ERG* and *HDAC1* expression in tumor specimens; Figure S8: HDAC inhibition leads to dose-dependent upregulation of NOTCH signaling genes *DLL1*, *NOTCH1*, and *HES1* and increased expression of H3K27ac. Table S1: Primer sequences, antibodies, Gateway Ref-Seqs, Luciferase Assay RefSeqs, vector maps; Table S2: Annotation sheet tissue microarray; Table S3: Predicted target genes and NCI pathways enriched for predicted target genes of *miR-449a*, *miR-449b-5p* and *miR-449c-5p*; Table S4: Gene set enrichment analysis results for hallmark gene sets positive or negative enriched in TCGA-PRAD T2E+; Table S5: BioCarta2016 pathway prediction for LNCaP T2E expression profiling; Table S6: Results co-immunoprecipitation HDAC1-FLAG in LNCaP cells, two replicates.
